# Mucopolysaccharidosis I, II, and VI: Brief review and guidelines for treatment

**DOI:** 10.1590/S1415-47572010005000093

**Published:** 2010-12-01

**Authors:** Roberto Giugliani, Andressa Federhen, Maria Verônica Muñoz Rojas, Taiane Vieira, Osvaldo Artigalás, Louise Lapagesse Pinto, Ana Cecília Azevedo, Angelina Acosta, Carmen Bonfim, Charles Marques Lourenço, Chong Ae Kim, Dafne Horovitz, Denize Bonfim, Denise Norato, Diane Marinho, Durval Palhares, Emerson Santana Santos, Erlane Ribeiro, Eugênia Valadares, Fábio Guarany, Gisele Rosone de Lucca, Helena Pimentel, Isabel Neves de Souza, Jordão Correa, José Carlos Fraga, José Eduardo Goes, José Maria Cabral, José Simionato, Juan Llerena, Laura Jardim, Liane Giuliani, Luiz Carlos Santana da Silva, Mara L. Santos, Maria Angela Moreira, Marcelo Kerstenetzky, Márcia Ribeiro, Nicole Ruas, Patricia Barrios, Paulo Aranda, Rachel Honjo, Raquel Boy, Ronaldo Costa, Carolina Souza, Flavio F. Alcantara, Silvio Gilberto A. Avilla, Simone Fagondes, Ana Maria Martins

**Affiliations:** 1 , Rede MPS BrasilBrazil; 2 Serviço de Genética Médica, Hospital de Clínicas de Porto Alegre, RSBrazil; 3 Grupo Hospitalar Conceição, Porto Alegre, RSBrazil; 4 Universidade Federal da Bahia, Salvador, BABrazil; 5 Hospital das Clínicas, Universidade Federal do Paraná, PRBrazil; 6 Escola de Medicina de Ribeirão Preto, Universidade de São Paulo, Ribeirão Preto, SPBrazil; 7 Instituto da Criança, Hospital de Clínicas, Universidade de São Paulo, SPBrazil; 8 Instituto Fernandes Figueira, Fundação Oswaldo Cruz, Rio de Janeiro, RJBrazil; 9 Hospital Universitário, Universidade de Brasília, DFBrazil; 10 Pontifícia Universidade Católica, Campinas, SPBrazil; 11 Serviço de Oftalmologia, Hospital de Clínicas de Porto Alegre, RSBrazil; 12 Universidade Federal do Mato Grosso do Sul, Campo Grande, MSBrazil; 13 Universidade Estadual de Ciências da Saúde, Maceió, ALBrazil; 14 Hospital Geral Albert Sabin, Fortaleza, CEBrazil; 15 Escola de Medicina, Universidade Federal de Minas Gerais, Belo Horizonte, MGBrazil; 16 Serviço de Fisiatria e Reabilitação, Hospital de Clínicas de Porto Alegre, RSBrazil; 17 Hospital Infantil Joana de Gusmão, Florianópolis, SCBrazil; 18 APAE, Salvador, BABrazil; 19 Universidade Federal do Pará, Belém, PABrazil; 20 Faculdade de Medicina, Universidade Federal do Rio Grande do Sul, Porto Alegre, RSBrazil; 21 Universidade Federal do Amazonas, Manaus, AMBrazil; 22 Hospital Infantil, Belo Horizonte, MGBrazil; 23 Departamento de Pediatria, Universidade Federal do Mato Grosso do Sul, Campo Grande, MSBrazil; 24 Hospital Infantil Pequeno Príncipe, Curitiba, PRBrazil; 25 Unidade de Fisiologia Pulmonar, Hospital de Clínicas, Porto Alegre, RSBrazil; 26 Hospital da Restauração, Recife, PEBrazil; 27 Instituto Martagão Gesteira, Universidade Federal do Rio de Janeiro, RJBrazil; 28 Serviço de Cardiologia, Hospital de Clínicas de Porto Alegre, RSBrazil; 29 Hospital Evangélico, Londrina, PRBrazil; 30 , Universidade Estadual do Rio de Janeiro, RJBrazil; 31 Serviço de Anestesiologia e Medicina Perioperativa, Hospital de Clínicas de Porto Alegre, RSBrazil; 32 , Sociedade Brasileira de Genética MédicaBrazil; 33 , Sociedade Brasileira de Patologia Clínica/Medicina LaboratorialBrazil; 34 , Associação Brasileira de Cirurgia PediátricaBrazil; 35 , Sociedade Brasileira de Pneumologia e TisiologiaBrazil; 36 Departamento de Pediatria, Universidade Federal de São Paulo, SPBrazil

**Keywords:** mucopolisaccharidoses, Hurler syndrome, Hunter syndrome, Maroteaux-Lamy syndrome, enzyme replacement therapy, treatment guidelines

## Abstract

Mucopolysaccharidoses (MPS) are rare genetic diseases caused by the deficiency of one of the lysosomal enzymes involved in the glycosaminoglycan (GAG) breakdown pathway. This metabolic block leads to the accumulation of GAG in various organs and tissues of the affected patients, resulting in a multisystemic clinical picture, sometimes including cognitive impairment. Until the beginning of the XXI century, treatment was mainly supportive. Bone marrow transplantation improved the natural course of the disease in some types of MPS, but the morbidity and mortality restricted its use to selected cases. The identification of the genes involved, the new molecular biology tools and the availability of animal models made it possible to develop specific enzyme replacement therapies (ERT) for these diseases. At present, a great number of Brazilian medical centers from all regions of the country have experience with ERT for MPS I, II, and VI, acquired not only through patient treatment but also in clinical trials. Taking the three types of MPS together, over 200 patients have been treated with ERT in our country. This document summarizes the experience of the professionals involved, along with the data available in the international literature, bringing together and harmonizing the information available on the management of these severe and progressive diseases, thus disclosing new prospects for Brazilian patients affected by these conditions.

Mucopolysaccharidoses (MPS) are a group of inborn errors of metabolism caused by a deficiency of specific lysosomal enzymes that affect glycosaminoglycan (GAG) catabolism. The accumulation of GAG in various organs and tissues of patients affected by MPS results in a series of signs and symptoms which make up a multisystemic clinical picture. To date, eleven enzyme defects that cause seven different types of MPS have been identified (Neufeld and Muenzer, 2001).

The participation of a multidisciplinary team of specialized professionals is recommended for the diagnosis, treatment, and monitoring of patients with MPS, because these diseases are rare and exhibit multisystemic involvement (Muenzer, 2004). A group of Brazilian professionals with experience in the treatment of MPS, representing all regions of the country, met to draft these guidelines for the treatment of MPS I, II, and VI, for which there currently is a specific therapy.

## General Information, Clinical Picture and Classification of MPS I, II, and VI

###  MPS I

Mucopolysaccharidosis type I (MPS I) is a chronic, progressive, multisystemic lysosomal disease caused by a deficiency or absence of activity of the α-L-iduronidase (IDUA) enzyme. Different mutations can cause variations in IDUA enzyme activity that are associated, in part, with the clinical variability observed over the course of the disease (Hirth *et al.*, 2007; Pastores *et al.*, 2007). MPS I, like the majority of lysosomal diseases, is inherited in an autosomal recessive manner and has an incidence of approximately 1 in 100,000 live births for the Hurler phenotype and up to 1 in 800,000 live births for the Scheie phenotype (Lowry *et al.*, 1990; Nelson, 1997; Meikle *et al.*, 1999; Poorthuis *et al.*, 1999; Neufeld and Muenzer, 2001).

The most common manifestations of MPS I include a characteristic facies, corneal clouding, macroglossia, hearing loss, hydrocephaly, cardiopathy, respiratory problems, hepatosplenomegaly, inguinal and umbilical hernia, dysostosis multiplex, limited joint mobility, and cognitive impairment. In addition, the accumulation of GAGs in rigid structures and paraspinal ligaments increases the potential for morbidity, resulting in major risks to the cervical column (Hite *et al.*, 2000; Weisstein *et al.*, 2004; Fuller *et al.*, 2005). Due to the involvement of various organs and tissues, patients with MPS I frequently require surgical interventions with a high rate of complications (Ard *et al.*, 2005).

MPS I is commonly classified into three clinical syndromes: Hurler, Hurler-Scheie, and Scheie. Because of the high variability of MPS I and the overlapping of symptoms in patients, it seems more appropriate to classify patients as having the attenuated form or the severe form (Vijay and Wraith, 2005). A review of this classification is currently under way.

**Severe form (Hurler syndrome):** This is the most severe MPS I phenotype (Soliman *et al.*, 2007), characterized by impaired cognitive development, progressive coarsening of facial features, hepatosplenomegaly, respiratory failure, cardiac valvulopathy, recurrent otitis media, corneal clouding, musculoskeletal manifestations such as joint stiffness and contractures, and dysostosis multiplex. The symptoms arise after birth and progress rapidly (Pastores *et al.*, 2007). Most of the patients with the severe phenotype which are not submitted to a specific treatment progress to death, on average, before the age of 10 years, due to complications related to brain damage or cardiorespiratory problems (Weisstein *et al.*, 2004; Boelens, 2006).

**Attenuated form (Hurler-Scheie syndrome):** This phenotype manifests in infancy, however with intermediate severity when compared with the Hurler phenotype. The somatic symptoms reduce life expectancy to the second or third decade of life (Pastores *et al.*, 2007; Soliman *et al.*, 2007). Generally, there is no cognitive impairment, but some patients may exhibit mild learning difficulties (Bjoraker *et al.*, 2006).

**Scheie syndrome:** This is the most attenuated form of MPS I (Soliman *et al.*, 2007), In which the symptoms occur later and progress slowly. Patients exhibit normal intelligence and survive until adulthood (Pastores *et al.*, 2007).

###  MPS II

Mucopolysaccharidosis II (MPS II or Hunter syndrome) is a rare genetic disease caused by deficiency of the lysosomal enzyme iduronate-2-sulfatase (IDS). MPS II has an incidence of approximately 0.31 to 0.71 per 100,000 live births (Nelson, 1997; Nelson *et al.*, 2003; Baenher *et al.*, 2005), and is found almost exclusively in young males because it is an X-linked condition. Recently, however, affected females – with a clinical picture in many cases similar to that of the young males – have been described (Tuschl *et al.*, 2005). MPS II is a chronic, progressive disease with a clinical picture similar in certain aspects to that of MPS I: there is great variability in the clinical manifestations, including central nervous system involvement, and can therefore be classified into a severe or “neuropathic” form and an attenuated or “non-neuropathic” form (Martin *et al.*, 2008; Wraith *et al.*, 2008).

Patients with MPS II exhibit upper respiratory tract dysfunctions, which can be classified as obstructive or restrictive (Sanjurjo-Crespo, 2007; Wraith *et al.*, 2008). These patients also experience a greater frequency of recurrent respiratory infections (Martin *et al.*, 2008). Another frequent complication, which also occurs in the other MPS types, is sleep apnea (Sanjurjo-Crespo, 2007; Martin *et al.*, 2008; Wraith *et al.*, 2008). With respect to musculoskeletal disorders, joint stiffness, pelvic dysplasia, and vertebral and rib abnormalities may be present (Sanjurjo-Crespo, 2007). Bone manifestations are called “dysostosis multiplex” ad exhibit specific characteristics in various bones (Martin *et al.*, 2008). Gastrointestinal tract manifestations include hepatomegaly, associated or not with splenomegaly (Wraith *et al.*, 2008). Umbilical and inguinal hernias are frequent findings as well (Sanjurjo-Crespo, 2007; Martin *et al.*, 2008; Schumacher *et al.*, 2008). Most patients develop recurrent otitis and virtually all will have some degree of hearing loss (Martin *et al.*, 2008). Dental abnormalities, as well as gingival hypertrophy and hyperplasia, may also be found in these patients (Martin *et al.*, 2008). Cardiologic manifestations are common and are usually observed at around 5 years of age, generally constituting the primary cause of death (Martin *et al.*, 2008). Ocular manifestations include papilledema, optic nerve atrophy, and retinal dystrophy (Anawis, 2006; Martin *et al.*, 2008; Schumacher *et al.*, 2008). Patients with MPS II also exhibit skin disorders, such as hirsutism (Wraith *et al.*, 2008), Mongolian spot, and papular lesions, caused by GAG deposits and considered typical of this type of MPS, although not exclusive to it (Ochiai *et al.*, 2003; Martin *et al.*, 2008; Wraith *et al.*, 2008).

From a neurological point of view, about two thirds of MPS II patients present with manifestations such as developmental delay and/or neurological regression (Schwartz *et al.*, 2007). These findings indicate the presence of the “neuropathic” form of the disease. More severely affected patients may experience seizures (Martin *et al.*, 2008), which sometimes manifest at the onset of the neurodegenerative picture. Behavioral changes, such as hyperactivity, aggressiveness, and obstinacy, may also be present in severely affected patients (Martin *et al.*, 2008). The attenuated (“non-neuropathic”) form is characterized by little or no central nervous system involvement, with preserved intelligence and an extended life expectancy. At times, classification is difficult, because there are patients with intermediate characteristics, such as early onset of respiratory problems, progressive upper airway obstruction, and compression of the vertebral column, among other signs and symptoms (Frossairt *et al.*, 2007; Sanjurjo-Crespo, 2007). Communicating hydrocephalus and spinal cord compression syndrome, as well as carpal tunnel syndrome, may also occur (Martin *et al.*, 2008).

###  MPS VI

Mucopolysaccharidosis VI (MPS VI or Maroteaux-Lamy syndrome) is a rare autosomal recessive genetic disease caused by deficiency of the enzyme *N-*acetylgalactosamine-4-sulfatase or arylsulfatase B (ARSB). The estimated incidence of MPS VI is 0.23 per 100,000 live births (Baenher *et al.*, 2005), but in Brazil preliminary data indicate that this incidence is higher (Coelho *et al.*, 1997; Albano *et al.*, 2000).

Patients with MPS VI exhibit a wide variability of multisystemic symptoms with a chronic and progressive course, where primarily the skeletal and cardiopulmonary systems, cornea, skin, liver, spleen, brain, and meninges are affected. The somatic involvement can resemble that of individuals with MPS I, but the patients' intelligence is usually normal. In general, patients have a short trunk and a thoracolumbar gibbus. Ocular manifestations include corneal clouding, glaucoma, pseudoglaucoma, and papilledema with optic atrophy in more advanced stages. Hypoacusia is the most common otological manifestation, generally associated with a conductive and neurosensory component. Respiratory involvement results from extrinsic and intrinsic alterations to the airways. A short neck, elevated epiglottis, deep cervical fossa, hypoplastic mandible, and tracheobronchomalacia contribute to the respiratory problems. Obstructive sleep apnea is also a frequent complication in MPS VI.

Although patients with MPS VI do not exhibit mental retardation as a direct consequence of the disease, their cognitive acquisitions may be impaired by the auditory and visual deficits and by the physical limitations inherent to the disease. Physical growth and development may be normal in the first years of life, stagnating at around six or eight years of age (Giugliani *et al.*, 2007). Cardiac involvement is a significant component of this disease and is responsible for a large part of the patients' morbidity and mortality (Tan *et al.*, 1992; Dilber *et al.*, 2002; Azevedo *et al.*, 2004; Oudit *et al.*, 2007a,b). Most of the individuals with MPS VI progress to death in their 2^nd^ or 3^rd^ decade of life, with heart failure, often secondary to chronic respiratory obstruction, as the primary cause (Harmatz *et al.*, 2004).

## Biochemical and Genetic Aspects

###  Laboratory diagnosis

A clinical suspicion of MPS constitutes grounds for performing a urinary GAG concentration determination. These concentrations are elevated in virtually all types of MPS, but the occurrence of normal levels is not reason enough to rule out this diagnosis in a patient with a suggestive clinical picture. Measurement of urinary GAG concentrations can be done by various methods. One recommended test is quantification by reaction with DMB (dimethylmethylene blue) solution. In contact with GAGs, DMB produces a compound whose absorbance can be measured at 520 nm, and the reaction is linear up to 70 μg/dL (De Jong *et al.*, 1989). The results can be expressed as mg GAGs/mg creatinine. Even though only 250 μL of urine are required for the reaction, a minimum of 2 mL should be sent to the laboratory (may be 24-h urine or a single random urine specimen). The urine should be kept frozen until the GAG concentration determination is performed. GAG levels in individuals with MPS are usually very elevated (three or more times) compared to normal levels. Urinary GAG excretion in normal individuals is higher at birth, decreasing rapidly thereafter (Iwata *et al.*, 2000); after the age of 21 years the concentration no longer changes. Therefore, the results must be interpreted according to the reference standards for each age bracket.

Chromatography or electrophoresis can be used to identify which type of GAG is present in excess (*e.g.*, dermatan sulfate, heparan sulfate, keratan sulfate), which helps define which enzymes should be tested initially (Leistner and Giugliani, 1998). A diagnosis of MPS should be confirmed via enzyme assay, documenting the deficient enzyme activity that is specific to each type of MPS. Any diagnostic test should be reviewed by a professional with experience in lysosomal diseases, since the assays are complex and the results are often difficult to interpret (Muenzer, 2004).

Identification of the genotype can be important for predicting the phenotype (and in some cases for therapeutic decisions), for allowing genetic family counseling, and for aiding in prenatal diagnosis. Therefore, it is necessary to obtain the DNA of the patient and/or a family member, which is generally extracted from blood, but may alternatively be obtained from oral mucosa cells, saliva, or other materials.

###  Genetic aspects

####  MPS I 

To date, approximately 100 mutations have been identified in the IDUA gene (Vijay and Wraith, 2005). Among these, W402X and Q70X have been associated with the severe form of the disease, the Hurler Syndrome (Fuller *et al.*, 2005). Described as null alleles, both are associated with undetectable production of the IDUA protein (Matte *et al.*, 2003). Besides these, two other less common mutations (R89Q and R89W) have been found in patients with the attenuated phenotype (Hein *et al.*, 2003). The relative frequency of the mutations considered to be prevalent seems to have a different pattern in Brazilian patients, possibly due to the greater miscegenation of our population, with implications for the molecular analysis protocols to be used in our country (Matte *et al.*, 2000; Pereira *et al.*, 2008).

Although molecular tests may determine the genotype, clinical and laboratory tests, which are useful for confirming the diagnosis, are not able to detect small differences in residual enzyme activity, thus making it impossible to predict the severity of the disease (Pastores *et al.*, 2007). Therefore, factors such as the age at onset of symptoms and the presence of two null mutations and of specific clinical characteristics (such as gibbus formation and delayed development) are important for a more precise classification of the disease (Pastores *et al.*, 2007).

####  MPS II 

MPS II is the only mucopolysaccharidosis with X-linked inheritance. The *IDS* gene is located at Xp28.1 and more than 300 mutations (including deletions, insertions, and substitutions) have been identified so far (Li *et al.*, 1999). However, a significant correlation between the type of mutation and the phenotype has not yet been established, although patients with total or partial deletion of the gene or with rearrangements between the gene and the pseudogene may exhibit a more severe phenotype. Moreover, it is interesting to observe that the same mutation can be associated with different phenotypes (Martin *et al.*, 2008).

####  MPS VI 

MPS VI is inherited in an autosomal recessive manner. The gene that codifies the enzyme arylsulfatase B (ARSB) is located on chromosome 5q13-14. The panel of mutations detected so far is fairly heterogeneous (Karageorgos *et al.*, 2007), with a low relative frequency of each mutation. Only in Portugal and in Brazil have relatively common mutations been identified (Petry *et al.*, 2003, 2005). A correlation between urinary GAG excretion and the clinical phenotype has now been established (Swiedler *et al.*, 2005), but there is no well-established correlation yet with the genotype of the affected individuals (Litjens *et al.*, 1996).

## Genetic Counseling and Prenatal Diagnosis

As genetic counseling provides the family with information regarding reproductive risks, it can contribute toward preventing the recurrence of MPS I, II, and VI. The risk of recurrence for a normal couple with a child affected by MPS I or VI, which are inherited in an autosomal recessive mode, is 25% for each new pregnancy. As in most autosomal recessive disorders, parental consanguinity is often present (Neufeld and Muenzer, 2001). In the case of MPS II, an X-linked condition, identification of female carriers is very important since, for each pregnancy, a female carrier has a 25% risk of having an affected child (50% risk for a male child). In families with a prior history of one of these types of MPS, prenatal diagnosis by means of chorionic villus biopsy or amniotic fluid collection during the first or second trimester of pregnancy, respectively, can detect further cases. The level of enzyme activity in the cells (by direct study or after culturing) leads to the diagnosis. Enzymatic diagnosis can be performed in umbilical cord blood, but the risks of the procedure and the gestational age at diagnosis are increased in this case. When mutations are already known in the family, this diagnosis may be quickly obtained by molecular analysis of the material collected (Rogoyski *et al.*, 1985).

## Treatment

Before the advent of hematopoietic stem cell transplant (HSCT) and especially of enzyme replacement therapy, the main focus of the treatment of MPS I, II, and VI was the prevention and management of complications. This treatment was symptomatic and palliative, based on a multidisciplinary team in which the participation of diverse medical specialties, such as cardiology, pulmonology, anesthesiology, orthopedics, physiatrics, otorhinolaryngology, ophthalmology, neurosurgery, etc., has been very important. This approach, aimed not only at providing treatment but also at promoting health, has been very important, even after the development of specific treatments. Physical therapists, occupational therapists, psychologists, and speech therapists are also essential in maintaining the health of these patients, preventing complications, and, to a certain degree, delaying the progression of the disease (Pastores *et al.*, 2007).

In the 1980s, the treatment of MPS with HSCT was proposed (Krivit, 2004; Lange *et al.*, 2006), and in the 1990s Enzyme Replacement Therapy (ERT) was developed, providing two therapeutic tools for restoring, at least partially, the activity of the deficient enzyme. ERT became a reality approved for clinical use in 2003 for MPS I, in 2005 for MPS VI, and in 2006 for MPS II (Kakkis *et al.*, 2001a,b; Wraith *et al.*, 2004, 2007; Harmatz *et al.*, 2005a,b, 2008; Wraith, 2005; Muenzer *et al.*, 2006, 2007; Sifuentes *et al.*, 2007; Clarke, 2008; Clarke *et al.*, 2009; Giugliani *et al.*, 2009).

###  Hematopoietic Stem Cell Transplantation (HSCT)

HSCT has been used in patients with mucopolysaccharidosis for the purpose of correcting the enzyme deficiency (Boelens *et al.*, 2007). Although it is a high-risk procedure with a high morbidity/mortality rate, many studies reveal that HSCT can, in fact, change the natural history of the disease, increasing life expectancy and improving many systemic abnormalities (Vellodi *et al.*, 1997; Wraith *et al.*, 2007). However, its indication still depends on the type of MPS, the patient's clinical picture, his/her age, and whether or not there is neurological impairment (McKinnis *et al.*, 1996; Aldenhoven *et al.*, 2008; Muenzer *et al.*, 2009).

####  MPS I 

The main indication of HSCT is for patients with the severe form of MPS I, because – if performed before two years of age – it seems to favorably and significantly alter their cognitive impairment (Boelens *et al.*, 2007; Muenzer *et al.*, 2009). Age is an important factor, since in our country many patients are diagnosed only after or close to the age of two years. In addition, to perform HSCT, a compatible donor is required, which may delay the procedure considerably, also reducing the potential benefits (Muenzer *et al.*, 2009). Another relevant aspect is the difficulty in predicting with certainty, at the onset of the disease, which patients will develop the severe form, making it hard to identify those for whom the risk-to-benefit ratio of HSCT would be favorable (Fuller *et al.*, 2005). Thus, despite international experience indicating that the potential benefit of HSCT is superior to that of ERT in patients with the severe form of MPS I when performed before two years of age, the difficulties mentioned above lead to HSCT being performed on a rather limited basis in Brazilian patients with MPS I - a reality that should be changed.

HSCT can halt progression of the neurological deficit, prevent premature death due to heart or liver disease, and prolong the survival of affected children. However, even when performed early, HSCT does not correct skeletal deformities, despite improving odontoid dysplasia and accelerating growth. Ophthalmologic abnormalities also improve significantly with HSCT. Pulmonary complications are frequent following transplantation, and their occurrence is related to several pre-transplant risk factors. There is evidence that ERT initiated around 12 weeks prior to transplant may reduce respiratory complications during the post-transplant period, which would be an indication for its use, although the follow-up time has not yet been long enough to permit assessment of the long-term impact of this combination (Tolar *et al.*, 2008). Graft-versus-host disease (GVHD) is also reported frequently, and various strategies have been used in the attempt to reduce this complication that greatly alters the patients' quality of life. The results of the transplants performed more recently show significant progress with this procedure and a survival rate of over 70% (Staba *et al.*, 2004; Boelens *et al.*, 2007; Aldenhoven *et al.*, 2008; Prasad *et al.*, 2008), however the rates obtained in the northern hemisphere cannot be automatically extended to Brazil, due to the different local conditions.

####  MPS II 

To date, the results of bone marrow transplants [BMT] in patients with MPS II have not been considered satisfactory (Martin *et al.*, 2008; Wraith *et al.*, 2008). However, encouraging developments have now been reported with HSCT performed very early in a limited number of MPS II patients (Martin *et al.*, 2006; Prasad *et al.*, 2008). In general, this therapy has not been recommended for these patients, due to the lack of clearly demonstrated neurological benefits and the high rate of morbidity and mortality (Zareba, 2007).

####  MPS VI 

BMT is considered a therapeutic alternative for MPS VI (Herskhovitz *et al.*, 1999), but ever since the introduction of ERT it has been relegated to a second place, because the risks of HSCT do not appear to exceed the benefits in this type of MPS, once patients do not have a cognitive deficit, and the systemic problems have responded satisfactorily to ERT without the risks of BMT (Giugliani *et al.*, 2007).

####  Outline of the transplantation protocol 

A patient with an indication for transplantation (in general, a patient under two years of age with the severe form of MPS I) should be referred to a BMT/HSCT reference unit capable of performing this type of procedure in these patients. Transplant shall be indicated only after a careful evaluation with respect to the basic disease and to prior complications, primarily pulmonary and neurological ones. A suitably compatible donor may be found among the members of the family or in national and international volunteer donor banks. Donors with greater compatibility and higher enzyme concentrations will be preferentially selected. The patient will undergo the protocol in use in the reference department. Following the infusion of stem cells, all supportive care measures will be maintained until the graft takes. During the severe pancytopenia period, broad-spectrum antibiotics, transfusions of irradiated blood products, total parenteral nutrition, and water-electrolyte replacement will be used. One month after the infusion of stem cells, graft acceptance will be confirmed by complete blood count, molecular biology techniques, and enzyme evaluation. The patient will be followed regularly at the transplant unit by means of enzyme concentration determinations, evaluation of graft acceptance, and monitoring with respect to post-transplant complications.

###  Enzyme Replacement Therapy (ERT)

Enzyme replacement therapy (ERT) is a treatment that consists of the periodic intravenous administration of the specific enzyme that is deficient in the patient. The first effective treatment with ERT performed in patients with Gaucher disease (Barton *et al.*, 1990) led to the search for a similar treatment for other lysosomal storage diseases. The first mucopolysaccharidosis treated with ERT was MPS I (Biomarin Pharmaceutical Inc), with ERT being subsequently approved for MPS VI (Biomarin Pharmaceutical Inc) and for MPS II (Shire HGT).

####  MPS I 

ERT for MPS I is performed by intravenous administration of laronidase, a protein analogous to human α-iduronidase produced by genetic engineering in a Chinese hamster ovary (CHO) cell expression system (Krivit, 2004). ERT with laronidase was approved for the treatment of patients in the United States in 2003 (Food and Drug Administration – FDA), in Europe in 2003 (European Medicines Agency – EMEA), and in Brazil in 2005 (National Health Surveillance Agency – ANVISA).

#####  Preclinical studies

Studies using the canine model of MPS I showed that intravenous administration of α-L-iduronidase exhibits somatic distribution and is able to reduce lysosomal accumulation in various tissues, with a decrease in liver GAG accumulation and in urinary GAG excretion after two weeks (Kakkis, 2002).

#####  Clinical studies

**Phase I/II -** Ten patients ranging in age from five to 22 years received 0.58 mg/kg of α-L-iduronidase intravenously once a week for 52 weeks (Kakkis *et al.*, 2001a).

Summary of the main study findings: (a) Hepatomegaly decreased significantly in all patients and liver size normalized in eight of the 10 patients as early as in the 26^th^ week; (b) The height and weight growth rate increased by an average of 85% and 131%, respectively, in the 52^nd^ week in six prepubescent patients; (c) The mean maximum motion range of shoulder flexion and elbow extension increased significantly; (d) The number of sleep apnea and hypopnea episodes decreased by 61%; (e) Heart function (evaluated by The New York Heart Association functional classification) improved by one or two classes in all patients; (f) Urinary GAG excretion decreased after three or four weeks of treatment; (g) Serum anti-α-L-iduronidase antibodies were detected in four patients.

**Phase II/III -** A randomized, double-blind, placebo-controlled, multinational study was performed, including 45 patients with MPS I (one with Hurler, 37 with Hurler-Scheie, and seven with Scheie), randomized to receive 0.58 mg/kg of either laronidase or placebo intravenously once a week for 26 weeks (Wraith *et al.*, 2004).

Summary of the main study findings: (a) After 26 weeks of treatment, the patients who received laronidase showed a mean improvement of 5.6 percentage points in the predicted normal Forced Vital Capacity (FVC) (median 3.0; p = 0.009) and 38.1 meters of distance in the Six-Minute Walking Test (6MWT) (median 38.5; p = 0.066; p = 0.039, analysis of covariance); (b) The use of laronidase also significantly reduced hepatomegaly and urinary GAG excretion; (c) In the more severely affected patients there was improvement in apnea/hypopnea and shoulder flexion; (d) Laronidase was well tolerated and practically all patients receiving the enzyme developed IgG antibodies, with no apparent clinical effect.

**Phase IV -** A prospective, open-label, multinational study that included 20 children (16 with Hurler syndrome and four with Hurler-Scheie syndrome), all under five years of age. All patients received intravenous treatment with 0.58 mg/kg or 1.16 mg/kg laronidase weekly for 52 weeks (Wraith *et al.*, 2007).

Summary of the main study findings: (a) Tolerance to laronidase was good with both dosages; (b) GAG levels decreased by approximately 50% in the 13^th^ week of treatment and 61.3% in the 52^nd^ week; (c) The liver edge decreased by 69.5% on palpation in those patients with a palpable liver at the time the study started; (d) The proportion of patients with left ventricular hypertrophy decreased from 53% to 17% in the 52^nd^ week; (e) A global assessment of the sleep studies revealed improvement or stabilization in 67% of the patients; (f) The apnea/hypopnea index decreased by 5.8 events per hour.

####  MPS II 

ERT for the treatment of MPS II is performed by intravenous administration of idursulfase, a glycosylated protein analogous to native human iduronate-2-sulfatase, produced by genetic engineering in a continuous human cell line (Muenzer *et al.*, 2007). ERT with idursulfase was approved for the treatment of patients in the United States in July 2006 (FDA), and in Europe in January 2007 (EMEA). In Brazil, registration with ANVISA occurred in 2008.

#####  Preclinical studies

The animal model used for MPS II was a mouse (IdS-KO) whose *IDS* gene had been modified by genetic engineering techniques. The study performed by Muenzer *et al.* (2002) demonstrated that IdS-KO mice already exhibited increased urinary GAG excretion at six weeks of age, and at 10 weeks of age they showed evidence of skeletal and facial abnormalities. GAG accumulation in the liver, kidneys, lungs, and heart valves was evident at all ages. Weekly doses of idursulfase (0.5 mg/kg) reduced urinary GAG excretion in these mice after the third infusion. The reduction in GAG in the liver, kidneys, heart, spleen, lungs, skin, and skeletal musculature was more pronounced in the animals treated with the 1 mg/kg dose. Another study (Garcia *et al.*, 2007) indicated that doses given weekly or every two weeks reduced urinary GAG excretion and hepatomegaly in the animals tested. These studies demonstrated that idursulfase was effective in reducing the level of GAGs in urine and tissue in mice.

#####  Clinical studies

**Phase I/II -** A double-blind study that included 12 patients aged 5 years or older, divided into three treatment groups. The groups received infusions of idursulfase every two weeks, at the following doses: 0.15, 0.50, and 1.50 mg/kg. The study duration was 27 weeks (Muenzer *et al.*, 2007).

Summary of the main study findings: (a) All patients treated with idursulfase, regardless of the dose, showed a reduction in mean urinary GAG excretion following the first infusion, with a faster decrease in the groups receiving the 0.5 and 1.5 mg/kg doses; (b) A reduction in liver and spleen volume occurred; (c) A significant increase in walk test distance (p = 0.013) was observed in the groups that received 0.50 and 1.50 mg/kg of idursulfase; (d) One year of treatment with idursulfase was well tolerated; (e) IgG antibodies were detected in 6/12 patients (three in the group that received 0.5 mg/kg and three in the group that received 1.5 mg/kg). The development of antibodies did not have any clinical or biological impact on idursulfase activity. None of the patients developed anti-idursulfase IgE antibodies.

**Phase II/III -** An international, multicenter study that included 96 patients ranging from five to 31 years of age, divided into three groups: placebo, idursulfase (0.5 mg/kg) once a week, and idursulfase (0.5 mg/kg) every two weeks. The duration of the study was 53 weeks. Randomization was stratified by age and by disease score at baseline (6MWT and FVC%) (Muenzer *et al.*, 2006).

Summary of the main study findings: (a) The combined variable (FVC% and 6MWT) score was significantly higher in the groups that received idursulfase; (b) After 53 weeks of weekly idursulfase infusions, the 6MWT distance increased significantly; (c) The predicted FVC increased in patients who received idursulfase weekly; (d) With respect to absolute FVC, there was a significant increase in the weekly idursulfase group; (e) Liver volume decreased by more than 20% after 18 weeks of treatment in both groups that received idursulfase; (f) About 80% of patients with hepatomegaly exhibited normal liver volume at between 18 and 53 weeks of treatment; (g) After 18 weeks of treatment, spleen volume decreased by approximately 20% to 25% in the groups that received idursulfase weekly and every other week, respectively; (h) After 53 weeks, spleen volume remained significantly reduced in the groups treated with idursulfase; (i) At week 53, GAG levels in the idursulfase groups were significantly lower. After 53 weeks of treatment, regardless of the idursulfase dosing regimen, 26/64 patients (40.6%) exhibited normal urine GAG levels, and the majority of patients were close to normal limits; (j) An improvement in elbow joint mobility was observed; (k) One year of treatment with idursulfase was well tolerated; (l) IgG antibodies were detected in 15 patients in the group that received idursulfase weekly and in 15 patients of the group that received idursulfase every two weeks; (m) IgM antibodies occurred in two patients, one in each idursulfase treatment group; (n) There was no impact on the central nervous system.

####  MPS VI 

ERT for the treatment of MPS VI is performed by intravenous administration of galsulfase, a recombinant form of the enzyme N-acetylgalactosamine 4-sulfatase, synthesized by means of genetic engineering from Chinese hamster ovary cells (Fuller *et al.*, 1998; Auclair *et al.*, 2003; Harmatz *et al.*, 2008). The marketing and use of galsulfase was approved in the United States in 2005 (FDA), in the European Union in January 2006 (EMEA), and was registered with ANVISA in February 2009.

#####  Preclinical studies

Studies using an experimental model of MPS VI (cats) showed that administration of galsulfase produced a significant improvement in some signs of the disease (Bielicki *et al.*, 1999; Turner *et al.*, 1999; Kakkis, 2002; Auclair *et al.*, 2003). They also showed a decrease in GAG storage in organs, an increase in joint mobility, and prevention or slowed progression of skeletal disease.

#####  Clinical studies

**Phase I/II -** The study by Harmatz *et al.* (2005b) was performed in six patients, using two different doses of the drug, 1 mg/kg and 0.2 mg/kg, given in weekly infusions during 48 weeks.

Summary of the main study findings: (a) The drug was well tolerated; (b) There was a reduction in GAG excretion via the urine.

**Phase II -** The study, performed in 10 patients, used the 1 mg/kg dose established in the previous study for 48 weeks, with weekly intravenous infusions (Harmatz *et al.*, 2005a).

Summary of the main study findings: (a) Confirmation of the results of the phase I/II study; (b) Improvement in the ability to climb stairs; (c) Improvement in the 12-minute walk test; (d) Feeling of improvement in joint stiffness and pain.

**Phase III -** The study used the same dose and administration method as the phase II study, but now with 39 patients for 24 weeks (Harmatz *et al.*, 2006).

Summary of the main study findings: (a) Confirmation of the results of the previous study; (b) Improvement in general resistance measured by means of the 12-minute walk test, and in the ability to climb stairs; (c) Reduction in urine GAG excretion; (d) Of the 54 patients who participated in these studies, only one did not develop specific antibodies to galsulfase.

## Guidelines For Enzyme Replacement Therapy

###  MPS I

The laronidase prescribing information approved by FDA (NDC 58468-70070-1) and EMEA in 2003, and registered in Brazil (ANVISA) in 2005, states that laronidase is indicated for patients with the Hurler and Hurler-Scheie forms of mucopolysaccharidosis type I and for patients with the Scheie form who exhibit moderate to severe symptoms. In Latin America, the only country that has currently published a consensus on the diagnosis and treatment of MPS I is Argentina (Argentine Pediatrics Society, 2008).

The inability of intravenously administered laronidase to reach the central nervous system, at least at the currently recommended dose of 0.58 mg/kg per week, limits its effects on neurological impairment in patients with the severe and neurodegenerative form of the disease (Hurler phenotype), therefore being indicated for the treatment of non-neurological symptoms of the disease.

The use of ERT in association with HSCT has not yet been established, although there is evidence that this combination reduces pulmonary complications following transplant (Tolar *et al.*, 2008). To date, the primary justification for defending the use of ERT in patients in whom HSCT is indicated is to improve the patient's physical condition while a compatible donor is sought (Wraith, 2001).

Objectively, ERT should be indicated in the following cases in which there is a confirmed diagnosis of MPS I: Patients of any age who are symptomatic and who exhibit at least one clinical manifestation that responds to treatment with ERT. These manifestations may be: (a) Respiratory diseases, such as upper airway obstructions, recurrent infection, restrictive diseases; (b) Cardiac disorders, such as cardiomyopathy and valve disease; (c) Osteoarticular disorders that impair locomotion or make it difficult, causing the patient to be dependent on other people for carrying out every-day activities; (d) Sleep apnea with an apnea index (AI) higher than one event/h of sleep for patients under 17 years of age, and higher than 5 events/h of sleep for adults; (e) Mean nocturnal O_2_ saturation < 92% in children and < 85% in adults; (f) Patients which are hard to intubate.

Drug characteristics and Usage Regimen (dose, frequency, and infusion time) for MPS I are presented in Table 1.

A recent study (Giugliani *et al.*, 2009) indicated that the administration of a double dose every other week does not result in significant disadvantages to the patient, and this administration regimen may be considered in cases in which a weekly infusion regimen is difficult to implement for some operational or logistical reason.

###  MPS II

ERT can be performed in all symptomatic patients with a confirmed MPS II diagnosis. Although Wraith *et al.* (2008) suggested that patients with significant CNS involvement should receive ERT for 12 to 18 months, and maintenance of ERT should be assessed after this period, the overall benefits of this treatment are questionable in patients with severe impairment of cognitive functions, since the intravenously administered enzyme does not cross the blood-brain barrier.

Objectively, ERT should be indicated in the following cases with a confirmed diagnosis of MPS II: Patients of any age who are symptomatic, who do not have severe cognitive impairment, and who exhibit at least one clinical manifestation that responds to treatment with ERT: (a) Respiratory diseases, such as upper airway obstructions, recurrent infections, restrictive diseases; (b) Osteoarticular disorders that impair locomotion or make it difficult, causing the patient to be dependent on other people for carrying out every-day activities; (c) Sleep apnea frequency higher than one event/h for patients under 18 years of age, and higher than 5 events/h for adults; (d) Mean nocturnal O_2_ saturation < 92% in children and < 85% in adults.

Although ERT has not been tested in clinical trials with patients under the age of 5 years, it has been used in small children in isolated cases, with no indications that the safety and efficacy profile are different from those observed in older children.

Drug characteristics and Usage Regimen (dose, frequency, and infusion time) for MPS II are presented in Table 1.

###  MPS VI

ERT may be administered to all symptomatic patients with a confirmed diagnosis of MPS VI, and is recommended as treatment of choice for this condition. Studies have demonstrated improvement in the walking test and in the ability to climb stairs (Harmatz *et al.*, 2006; Giugliani *et al.*, 2007), improvement in MPS VI-related bone disease, as well as improvement in growth pattern in a patient treated as of the eighth week of life (McGill *et al.*, 2009). It is known, however, that some tissues, such as the cornea, due to their reduced perfusion, and the central nervous system, due to the blood-brain barrier, are not significantly affected by the action of the intravenously administered enzyme (Giugliani *et al.*, 2007; Clarke, 2008).

Objectively, ERT should be indicated in the following cases with a confirmed diagnosis of MPS VI: Patients of any age who are symptomatic and have at least one clinical manifestation that responds to treatment with ERT. These manifestations may be: (a) Respiratory diseases, such as upper airway obstructions, recurrent infections, restrictive diseases; (b) Osteoarticular disorders that impair locomotion or make it difficult, causing the patient to be dependent on other people for carrying out every-day activities; (c) Sleep apnea frequency higher than 1 event/h for patients under 18 years of age, and higher than 5 events/h for adults; (d) Mean nocturnal O_2_ saturation < 92% in children and < 85% in adults; (e) Patients who are hard to intubate.

Drug characteristics and Usage Regimen (dose, frequency, and infusion time) for MPS VI are presented in Table 1.

## Other Information Common To The Handling, Preparation, and Administration of Laronidase, Idursulfase, and Galsulfase

**Usage Regimen -** (a) Use of the standard dose is recommended. Some small adjustments may be made, as long as the dose used does not vary more than 10% in relation to the standard dose. Similarly, the final monthly dose should not vary more than 10% with regard to the ideal monthly dose, established according to the standard dose. (b) Dose calculation should be reviewed every three months, whether the patients are children or adults. (c) It is recommended that the infusion be initially administered in a hospital environment and preferably in a bright environment that is pleasant for the patient. Given the increasing number of patients throughout the country who are receiving ERT, it is recommended that this procedure be standardized within the Brazilian Integrated Health System (SUS), so as to become one of the procedures officially considered to be performed in a “day hospital” setting. (d) It is important to alternate the peripheral vein puncture sites. Whenever a totally implanted central catheter is used, use of EMLA® is recommended (1 h or 1 h 30 min pre-puncture). (e) The patient should be observed for at least 1 h after the end of the infusion, at least during the first three months of treatment, if it is not possible to do so for the ideal period, which is six months. After this observation period, if there is no complicating factor, the patient may be released immediately following the infusion.

**Contraindications -** ERT is not indicated for women who are pregnant or nursing, unless it is absolutely essential. Terminal patients should not receive ERT either, nor should patients with a severe concomitant disease, the prognosis of which will not change as a result of the ERT.

**Premedication -** Possible infusion reactions are very specific to each patient, so the physician should assess the need for premedication and its strength on a case-by-case basis. Premedication with antipyretics and/or antihistamines is generally used for ERT in patients with MPS I. For patients with MPS VI receiving ERT, antihistamines have been used, with or without antipyretics, about 1 h prior to the start of the infusion. If there is an infusion reaction that persists even with the use of antipyretics and antihistamines, the use of corticosteroids prior to ERT should be considered, *e.g.*, prednisolone (1 mg/kg), 12 h and 1 h before the infusion. The use of premedication is not routinely prescribed in MPS II patients receiving ERT, except for preventing recurrence of infusion reactions.

**Drug Preparation -** Using proper asepsis techniques, the drug should be prepared as follows: (a) Determine the number of vials to be diluted, based on the patient's weight and the standard recommended dose of the replacement enzyme, adjusting it in such a way that whole vials are used; (b) Remove the vials from the refrigerator, to allow them to reach room temperature. These vials should not be heated; (c) The solution is transparent or somewhat yellowish, and clear or slightly opalescent, as some transparent particles may be present. If these characteristics of the solution are altered, these vials should not be used; (d) Determine the total final volume to be infused, which depends on the patient's weight and the drug to be prepared: MPS I: 100 mL (weight ≤ 20 kg) or 250 mL (weight > 20 kg); MPS II: 100 mL (for all weights) plus the total calculated volume of idursulfase; MPS VI: 250 mL (in general – for weights less than or equal to 20 kg; in patients who are susceptible to volume overload, the physician may consider the total volume of 100 mL); (e) Slowly aspirate the calculated volume of enzyme from the vials, taking care not to shake the solution, since shaking can denature the product and render it biologically inactive; (f) From the corresponding bag of physiological saline solution (100 mL or 250 mL), remove a volume equal to that calculated and aspirated from the vials of enzyme, so that, after adding the volume of enzyme, the total final volume of 100 mL or 250 mL, is reconstituted (this step is not necessary for idursulfase, since the orientation in the prescribing information is to dilute the total calculated volume of idursulfase in 100 mL of 0.9% Sodium Chloride Injection); (g) The addition of the enzyme solution to the bag of physiological saline solution has to be slow, and the bag containing the final solution has to be rotated gently, to permit homogeneous distribution of the drug; (h) This solution should be used immediately. If immediate use is not possible, the solution must be stored under refrigeration (2 °C to 8 °C) for a maximum period of 36 h from preparation to the end of administration of the solution (24 h for idursulfase, according to the Brazilian product information). Do not leave the prepared solution at room temperature; (i) In the case of MPS I, the use of albumin is recommended in the United States, but it is not used in the European countries. In Brazil, the ANVISA-approved prescribing information also recommends its use. However, the experience of Brazilian specialists indicates that the use of albumin can be dispensed with.

**Infusion Rate -** After preparation of the drug, the infusion should be administered in an incrementally increasing manner as recommended below. However, in the event of reactions associated with the infusion, these incrementally increased rates and the final maximum rate reached may be modified according to each patient's tolerance.

MPS I: (a) Weight less than or equal to 20 kg (total volume 100 mL): 2 mL/h x 15 min; 4 mL/h x 15 min; 8 mL/h x 15 min; 16 mL/h x 15 min; 32 mL/h x ~3 h; (b) Weight more than 20 kg (total volume 250 mL): 5 mL/h x 15 min; 10 mL/h x 15 min; 20 mL/h x 15 min; 40 mL/h x 15 min; 100 mL/h x ~3 h.

MPS II: 8 mL/h x 15 min; 16 mL/h x 15 min; 24 mL/h x 15 min; 32 mL/h x 15 min; 40 mL/h x ~2 h. This rate may be increased by 8 mL/h x 15 min, without exceeding the maximum rate of 100 mL/h.

MPS VI: 6 mL/h x 1 h; 80 mL/h x ~3 h.

**Use of Filters -** It is recommended that the administration of laronidase, idursulfase, and galsulfase solution be performed using an infusion set with a 0.2 μm filter.

**Adverse Reactions – Conduct -** The infusion reactions most commonly reported with the use of ERT were: pyrexia, headache, abdominal pain, dyspnea, chills, arthralgia, pruritus, hypertension/hypotension, urticaria, and exanthema (rash). If an infusion reaction occurs, regardless of whether premedication was used, the following measures should be taken, in this order, until the symptoms improve: reduction of the infusion rate, temporary discontinuation of the infusion, additional administration of antipyretics and antihistamines.

If a severe hypersensitivity reaction or an anaphylactic reaction occurs, the infusion should be stopped immediately and appropriate supportive measures should be promptly taken, according to the picture presented. The use of corticosteroids and airway and venous access maintenance measures may be necessary, and resuscitation measures must be implemented in extreme cases. For this reason, it is recommended that the infusion center should have the equipment necessary for comprehensive care of cardiorespiratory arrest (crash cart) and have easy access to the emergency room.

If the use of epinephrine is considered, it should be used with extreme caution, due to the increased prevalence of coronary disease in many patients with MPS.

The risk-to-benefit ratio of enzyme administration following a severe hypersensitivity reaction or anaphylactic reaction should be evaluated and, if ERT infusions are reinitiated, appropriate resuscitation measures should be available for use in extreme cases.

Ideally, before initiation of ERT, blood should be drawn for antibody level determination. This sample shall be kept until this determination is necessary, *i.e.*, in the event the patient experiences an infusion reaction. If the patient does experience an infusion reaction, blood should be drawn again between 1 and 2 h from the onset of the reaction, or according to the enzyme manufacturer's directions.

**Adverse Reactions – Pharmacovigilance -** Any side-effect should be reported as soon as possible to the Pharmacovigilance Department of ANVISA and to the pharmacovigilance section of the hospital, if applicable. In addition, the companies responsible for the drugs laronidase (Genzyme), idursulfase (Shire/HGT), and galsulfase (BioMarin) request that they be notified via their medical departments, for pharmacovigilance purposes.

**Clinical Routine -** Before the start of each infusion, a brief history should be taken and a targeted physical examination, including the checking of vital signs, should be performed. The collection of samples for monitoring tests may be indicated. Patients do not need to be fasting nor have their diets modified because of the infusion.

**Criteria for Discontinuation of Treatment -** To date, there are no established criteria determining the indication for discontinuation of treatment, however it is recommended that ERT be discontinued: (a) During pregnancy and breastfeeding; (b) In patients who, despite ERT, have progressed to terminal disease or experience a significant worsening of their clinical parameters, measured at least every six months and preferably over a period of at least 12 months of ERT; (c) In patients who do not exhibit any measurable clinical benefit, taking into consideration the natural rate of progression of the disease, based on parameters measured at least every six months and preferably over a period of at least than 12 months of ERT.

The possibility of discontinuation of treatment should be mentioned to the parents/patient or legal guardians when ERT is being considered and prior to its initiation. During clinical monitoring of a patient receiving ERT, the ERT therapeutic response parameters should be evaluated periodically and discussed with the parents/patient or legal guardians. If discontinuation of ERT is being considered, this should be discussed with the parents/patient or legal guardians.

When temporary suspension of ERT is considered, it should be taken into account that the few reports on ERT interruption found in the literature show that discontinuation of this treatment can lead to a rapid deterioration of the patient's clinical picture (Anbu *et al.*, 2006; Wegrzyn *et al.*, 2007).

**Presymptomatic Treatment -** Although there are fairly encouraging results, the benefits of presymptomatic treatment observed in various case reports have not yet been assessed via clinical trials (which are currently under way in the case of MPS VI). Thus, in cases in which the physician considers it to be indicated, the treatment of MPS I, MPS II, and MPS VI prior to the onset of symptoms should be presented to the family as an experimental procedure, and it is suggested that an Informed Consent Form approved by the competent ethical bodies be utilized.

**Treatment in Children Under Five Years of Age -** The use of laronidase in children under five years of age has been shown to be safe, as demonstrated in a specific clinical study in small children with MPS I (Wraith *et al.*, 2007). This favorable result in terms of safety has also been consistently observed in several cases of young MPS II and MPS VI patients treated with ERT (Kim *et al.*, 2008), although it has not yet been formally assessed in small children via clinical trials.

**Alternative Routes of Administration -** Brazil was a pioneer in the intrathecal administration of recombinant enzyme in a patient with MPS I, for treatment of spinal cord compression. This experience had encouraging results and was reported in the literature (Muñoz-Rojas *et al.*, 2008). Additional cases of Brazilian patients with MPS and spinal cord compression (one with MPS I and another with MPS VI) were similarly treated and the reports are being prepared for publication. However, intrathecal administration of the enzyme should be considered an experimental procedure for the time being.

**Home Infusion -** Home infusion may constitute an option for patients who, after three to six months of hospital infusion, have not experienced any significant infusion reactions. It is recommended that both the infusion location and the drug storage and preparation location be approved by the person in charge of the reference center's medical staff, and that a professional nurse trained for this specific procedure monitor the infusion all the time and regularly inform the reference center about the procedure. The patient undergoing home infusion must have regular medical checkups at the reference center at least every three months (Cox-Brikman *et al.*, 2007).

## Prospects and Conclusions

The authors of this study are convinced that a better future for patients suffering from mucopolysaccharidoses depends on the proper identification, understanding and management of the multisystemic manifestations of these diseases, including supportive measures (which should be part of the regular multidisciplinary care of these patients) and specific therapies. There are indications that earlier detection and treatment of patients, possibly by means of newborn screening, may contribute to a better prognosis. A definitive cure may perhaps be achieved through gene therapy, but this moment could still take some time to arrive.

Although inhibition of glycosaminoglycan synthesis and the restoration of enzyme activity with small molecules may also come to play a role in the management of MPS, the main advance currently available is ERT. Along with HSCT (for specific situations), ERT has enabled a radical change in the panorama of treatment for mucopolysaccharidosis I, II, and VI in the past decade and is helping to provide a better understanding of the physiopathology of the disease (Pereira *et al.*, 2008) and potential biomarkers (Randall *et al.*, 2008). It is further possible that its benefits may be extended to MPS IV A shortly, with prospects for the treatment of MPS III A and of the cognitive deficit in MPS II via administration of the enzyme directly into the central nervous system (CNS).

Presently, a large number of Brazilian centers, including departments in all regions of the country, have already some experience with ERT for MPS I, II, and VI, acquired not only by treating patients, but also through the participation of some groups in clinical trials involving ERT for these conditions. Taking the three types of MPS together, over 200 patients have been treated with ERT in our country so far. The experience of professionals, along with the data available in the international literature, enabled the drafting of this document, produced with the purpose of joining and harmonizing the information available on the treatment of these severe and progressive diseases, which are, fortunately, treatable today, offering new prospects for Brazilian patients affected by these conditions.

## Supplementary Material

The following online material is available for this article:

Note about the steps followed for the drafting of this document

This material is made available as part of the online article from http://www.scielo.br.gmb.

## Figures and Tables

**Table 1:** Characteristics of the Drug and Usage Regimen (dose, frequency, and infusion time) for MPS I., MPS II and MPS VI.

	Mucopolysaccharidosis I	Mucopolysaccharidosis II	Mucopolysaccharidosis VI
Drug and manufacturer	Aldurazyme® (Genzyme Corporation)	Elaprase® (Shire HGT)	Naglazyme® (BioMarin Pharmaceuticals)
How supplied	Vials containing 2.9 mg/5 mL (0.58 mg/mL)	Vials containing 6 mg/3 mL (2 mg/mL)	Vials containing 5 mg/5 mL (1 mg/mL)
Standard dose and route of administration	0.58 mg/kg, intravenously	0.50 mg/kg, intravenously	1 mg/kg, intravenously
Frequency	Weekly (7 ± 3 days)	Weekly (7 ± 3 days)	Weekly (7 ± 3 days)
Infusion time	Approximately 3-4 h	From 1 to 3 h	A minimum of 4 h

## References

[Albanoetal2000] Albano L.M., Sugayama S.S., Bertola D.R., Andrade C.E., Utagawa C.Y., Puppi F., Nader H.B., Toma L., Coelho J., Leistner S. (2000). Clinical and laboratorial study of 19 cases of mucopolysaccharidoses. Rev Hosp Clin Fac Med São Paulo.

[Aldenhovenetal2008] Aldenhoven M., Boelens J.J., De Koning T.J. (2008). The clinical outcome of Hurler syndrome after stem cell transplantation. Biol Blood Marrow Transplant.

[Anawis2006] Anawis M.A. (2006). Hunter syndrome (MPS II-B): A report of bilateral vitreous floaters and maculopathy. Ophthalmic Genet.

[Anbuetal2006] Anbu A., Mercer J., Wraith J.E. (2006). Effect of discontinuing of laronidase in a patient with mucopolysaccharidosis type I. J Inherit Metab Dis.

[Ardetal2005] Ard J.L., Bekker A., Frempong-Boadu A.K. (2005). Anesthesia for an adult with mucopolysaccharidosis I. J Clin Anesth.

[ArgentinePediatricsSocietySubcommissionsCommitteesWorkingGroups2008] Argentine Pediatrics SocietySubcommissions Committees Working Groups (2008). Consensus on mucopolysaccharidosis type I diagnosis and treatment. Arch Argent Pediatr.

[Auclairetal2003] Auclair D., Hopwood J.J., Brooks D.A., Lemontt J.F., Crawley A.C. (2003). Replacement therapy in Mucopolysaccharidosis type VI: Advantages of early onset of therapy. Mol Genet Metab.

[Azevedoetal2004] Azevedo A.C., Schwartz I.V., Kalakun L., Brustolin S., Burin M.G., Beheregaray A.P., Leistner S., Giugliani C., Rosa M., Barrios P. (2004). Clinical and biochemical study of 28 patients with mucopolysaccharidosis type VI. Clin Genet.

[Baenheretal2005] Baenher F., Schmiedeskamp C., Krummernauer F., Miebach E., Bajbouj M., Whybrac C., Miebach E., Bajbouj M., Whybra C., Kohlschütter A. (2005). Cumulative incidence rates of the mucopolysaccharidoses in Germany. J Inherit Metab Dis.

[Bartonetal1990] Barton N.W., Furbish F.S., Murray G.J., Garfield M., Brady R.O. (1990). Therapeutic response to intravenous infusions of glucocerebrosidase in a patient with Gaucher disease. Proc Natl Acad Sci USA.

[Bielickietal1999] Bielicki J., Crawley A.C., Davey R.C., Varnai J.C., Hopwood J.J. (1999). Advantages of using same species enzyme for replacement therapy in a feline model of mucopolysaccharidosis type VI. J Biol Chem.

[Bjorakeretal2006] Bjoraker K.J., Delaney K., Peters C., Krivit W., Shapiro E.G. (2006). Long term-outcomes of adaptive functions for children with mucopolysaccharidosis I (Hurler Syndrome) treated with hematopoietic stem cell transplantation. J Dev Behav Pediatr.

[Boelens2006] Boelens J.J. (2006). Trends in haematopoietic cell transplantation for inborn errors of metabolism. J Inherit Metab Dis.

[Boelensetal2007] Boelens J.J., Wynn R.F., O'meara A., Veys P., Bertrand Y., Souillet G., Wraith J.E., Fischer A., Cavazzana-Calvo M., Sykora K.W. (2007). Outcomes of hematopoietic stem cell transplantation for Hurler's syndrome in Europe: A risk factor analysis for graft failure. Bone Marrrow Transplant.

[Clarke2008] Clarke L.A. (2008). Idursulfase for the treatment of mucopolysaccharidosis II. Expert Opin Pharmacother.

[Clarkeetal2009] Clarke L.A., Wraith J.E., Beck M., Kolodny E.H., Pastores G.M., Muenzer J., Rapoport D.M., Berger K.I., Sidman M., Kakkis E.D. (2009). Long-term efficacy and safety of laronidase in the treatment of mucopolysaccharidosis I. Pediatrics.

[Coelhoetal1997] Coelho J.C., Wajner M., Burin M.G., Vargas C.R., Giugliani R. (1997). Selective screening of 10,000 high-risk Brazilian patients for the detection of inborn errors of metabolism. Eur J Pediatr.

[Cox-Brikmanetal2007] Cox-Brikman J., Timmermans R.G.M., Wijbrug F.A., Donker W.E., van de Ploeg A.T., Aerts J.M., Hollak C.E. (2007). Home treatment with enzyme replacement therapy for mucopolysaccharidosis type I is feasible and safe. J Inherit Metab Dis.

[DeJongetal1989] De Jong J.G., Wevers R.A., Laarakkers C., Poorthuis B.J. (1989). Dimethylmethylene blue-based spectrophotometry of glycosaminoglycans in untreated urine: A rapid screening procedure for mucopolysaccharidoses. Clin Chem.

[Dilberetal2002] Dilber E., Celiker A., Karagöz T., Kalkanoglu H.S. (2002). Permanent transfemoral pacemaker implantation in a child with Maroteaux Lamy syndrome. Pacing Clin Electrophysiol.

[Frossairtetal2007] Frossairt R., Moreira da Silva I., Maire I. (2007). Mucopolysaccharidosis type II: An update on mutation spectrum. Acta Paediatr.

[Fulleretal1998] Fuller M., Hopwood J.J., Anson D.S. (1998). Receptor mediated binding of two glycosylation of N-acetylgalactosamine-4-sulphatase. Biochim Biophys Acta.

[Fulleretal2005] Fuller M., Brooks D.A., Evangelista M., Hein L.K., Hopwood J.J., Meikle P.J. (2005). Prediction of neuropathology in mucopolysaccharidosis I patients. Mol Genet Metab.

[Garciaetal2007] Garcia A.R., Pan J., Lamsa J.C., Muenzer J. (2007). The characterization of a murine model of mucopolysaccharidosis II (Hunter syndrome). J Inherit Metab Dis.

[Giuglianietal2007] Giugliani R., Harmatz P., Wraith J.E. (2007). Management guidelines for mucopolysaccharidosis VI. Pediatrics.

[Giuglianietal2009] Giugliani R., Muñoz-Rojas M.V., Martins A.M., Valadares E.R., Clarke J.T., Góes J.E., Kakkis E.D., Worden M.A., Sidman M., Cox G.F. (2009). A dose-optimization trial of laronidase (Aldurazyme®) in patients with mucopolysaccharidosis I. Mol Genet Metabol.

[Harmatzetal2004] Harmatz P., Whitley C.B., Waber L., Pais R., Steiner R., Plecko B., Kaplan P., Simon J., Butensky E., Hopwood J.J. (2004). Enzyme replacement therapy in mucopolysaccaridosis VI (Maroteaux-Lamy Syndrome). J Pediatr.

[Harmatzetal2005a] Harmatz P., Ketteridge D., Giugliani R., Guffon N., Teles E.L., Miranda M.C., Yu Z.F., Swiedler S.J., Hopwood J.J. (2005a). Direct comparison of measures of endurance, mobility, and joint function during enzyme-replacement therapy of Mucopolysaccharidosis VI (Maroteaux-Lamy Syndrome): Results after 48 weeks in a phase 2 open-label clinical study of recombinant human N-acetylgalactosamine 4-sulfatase. Pediatrics.

[Harmatzetal2005b] Harmatz P., Kramer W.G., Hopwood J.J., Simon J., Butensky E., Swiedler S.J. (2005b). Pharmacokinetic profile of recombinant human N-acetylgalactosamine 4-sulphatase enzyme replacement therapy in patients with mucopolysaccharidosis VI (Maroteaux-Lamy syndrome): A phase I/II study. Acta Paediatr Suppl.

[Harmatzetal2006] Harmatz P., Giugliani R., Schwartz I., Guffon N., Teles E., Sá Miranda M. (2006). Enzyme replacement therapy for mucopolysaccharidosis VI: A phase 3, randomized, double-blind, placebo-controlled, multinational study of recombinant human N-acetylgalactosamine 4-sulfatase (recombinant human arylsulfatase B or rhASB) and follow-on, open-label extension study. J Pediatr.

[Harmatzetal2008] Harmatz P., Giugliani R., Schwartz I., Guffon N., Teles E., Sá Miranda M., Wraith J.E., Beck M., Arash L., Scarpa M. (2008). Long-term follow-up of endurance and safety outcomes during enzyme replacement therapy for mucopolysaccharidosis VI: Final results of three clinical studies of recombinant human N-acetylgalactosamine 4-sulfatase. Mol Genet Metab.

[Heinetal2003] Hein L.K., Hopwood J.J., Clements P.R., Brooks D.A. (2003). The alpha-L-iduronidase mutations R89Q and R89W result in an attenuated mucopolysaccharidosis type I clinical presentation. Biochim Biophys Acta.

[Herskhovitzetal1999] Herskhovitz E., Young E., Rainer J., Hall C.M., Lidchi V., Chong K., Vellodi A. (1999). Bone marrow transplantation for Maroteaux-Lamy syndrome (MPS VI): Long-term follow-up. J Inherit Metab Dis.

[Hirthetal2007] Hirth A., Berg A., Greve G. (2007). Successful treatment of severe heart failure in an infant with Hurler syndrome. J Inherit Metab Dis.

[Hiteetal2000] Hite S.H., Peters C., Krivit W. (2000). Correction of odontoid dysplasia following bone-marrow transplantation and engraftment (in Hurler syndrome MPS 1H). Pediatr Radiol.

[Iwataetal2000] Iwata S., Sukegawa K., Kokuryu M., Tomatsu S., Kondo N., Iwasa S., Orii T. (2000). Glycosaminoglycans in neonatal urine. Arch Dis Child Fetal Neonatal Ed.

[Kakkis2002] Kakkis E.D. (2002). Enzyme replacement therapy for the mucopolysaccharide storage disorders. Expert Opin Investig Drugs.

[Kakkisetal2001a] Kakkis E.D., Muenzer J., Tiller G.E., Waber L., Belmont J., Passage M., Izykowski B., Phillips J., Doroshow R., Walot I. (2001a). Enzyme Replacement Therapy in Mucopolysaccharidosis I. N Engl J Med.

[Kakkisetal2001b] Kakkis E.D., Schuchman E., He X., Wan Q., Kania S., Wiemelt S., Hasson C.W., O'Malley T., Weil M.A., Aguirre G.A. (2001b). Enzyme replacement therapy in feline mucopolysaccharidosis I. Mol Genet Metab.

[Karageorgosetal2007] Karageorgos L., Brooks D.A., Pollar A., Melville E.L., Hein L.K., Clements P.R., Ketteridge D., Swiedler S.J., Beck M., Giugliani R. (2007). Mutational analysis of 105 mucopolysaccharidosis type VI patients. Hum Mutat.

[Kimetal2008] Kim K.H., Decker C., Burton B.K. (2008). Successful management of difficult infusion-associated reactions in a young patient with mucopolysaccharidosis type VI receiving recombinant human arylsulfatase B (galsulfase [Naglazyme]). Pediatrics.

[Krivit2004] Krivit W. (2004). Allogeneic stem cell transplantation for the treatment of lysosomal and peroxisomal metabolic diseases. Springer Sem Immunopathol.

[Langeetal2006] Lange M.C., Teive H.A., Troiano A.R., Bitencourt M., Funke V.A., Setúbal D.C., Zanis J., Medeiros C.R., Werneck L.C., Pasquini R. (2006). Bone marrow transplantation in patients with storage diseases: A developing country experience. Arq Neuro-Psiquiatr.

[LeistnerandGiugliani1998] Leistner S., Giugliani R. (1998). A useful routine for the biochemical detection and diagnosis of mucopolysaccharidoses. Genet Mol Biol.

[Lietal1999] Li P., Bellows A.B., Thompson J.N. (1999). Molecular basis of iduronate-2-sulphatase gene mutations in patients with mucopolysaccharidosis type II (Hunter syndrome). J Med Genet.

[Litjensetal1996] Litjens T., Brooks D.A., Peters C., Gibson G.J., Hopwood J.J. (1996). Identification, expression, and biochemical characterization of N-acetylgalactosamine-4-sulfatase mutations and relationship with clinical phenotype in MPS-VI patients. Am J Hum Genet.

[Lowryetal1990] Lowry R.B., Applegarth D.A., Toone J.R., MacDonald E., Thunem N.Y. (1990). An update on the frequency of mucopolysaccharide syndromes in British Columbia. Hum Genet.

[Martinetal2006] Martin P.L., Carter S.L., Kernan N.A., Sabdev I., Wall D., Pietryga D., Wagner J.E., Kurtzberg J. (2006). Results of the Cord Blood Transplantation Study (COBLT): Outcomes of unrelated donor umbilical cord blood transplantation in pediatric patients with lysosomal and peroxisomal storage diseases. Biol Blood Bone Marrow Transplant.

[Martinetal2008] Martin R., Beck M., Eng C., Giugliani R., Harmatz P., Muñoz Vand Muenzer J. (2008). Recognition and diagnosis of mucopolysaccharidosis II (Hunter Syndrome). Pediatrics.

[Matteetal2000] Matte U., Leistner S., Lima L., Schwartz I., Giugliani R. (2000). Unique frequency of known mutations in Brazilian MPS I patients. Am J Med Genet.

[Matteetal2003] Matte U., Yogalingam G., Brooks D., Leistner S., Schwartz I., Lima L. (2003). Identification and characterization of 13 new mutations in mucopolysaccharidosis type I patients. Mol Genet Metab.

[McGilletal2009] McGill J.J., Inwood A.C., Coman D.J., Lipke M.L., de Lore D., Swiedler S.J., Hopwood J.J. (2009). Enzyme replacement therapy for mucopolysaccharidosis VI from 8 weeks of age – A sibling control study. Clin Genet.

[McKninkkisetal1996] McKninkkis E.J.R., Sulzbacher S., Rutledege J.C., Sanders J., Scott C. (1996). Bone marrow transplantation in Hunter syndrome. J Pediatr.

[Meikleetal1999] Meikle P.J., Hopwood J.J., Clague A., Carey W.F. (1999). Prevalence of lysosomal storage disorders. JAMA.

[Muenzer2004] Muenzer J. (2004). The mucopolysaccharidoses: A heterogeneous group of disorders with variable pediatric presentations. J Pediatr.

[Muenzeretal2002] Muenzer J., Lamsa J.C., Garcia A., Da Costa J., Garcia J., Treco D.A. (2002). Enzyme replacement therapy in mucopolysaccharidosis type II (Hunter syndrome): A preliminary report. Acta Paediatr Suppl.

[Muenzeretal2006] Muenzer J., Wraith J.E., Beck M., Giugliani R., Harmatz P., Eng C.M., Vellodi A., Martin R., Ramaswami U., Gucsavas-Calikoglu M. (2006). A phase II/III clinical study of enzyme replacement therapy with idursulfase in mucopolysaccharidosis II (Hunter syndrome). Genet Med.

[Muenzeretal2007] Muenzer J., Guscsavas-Calikoglu M., Shawn E., Schuetz T.J., Kimura A. (2007). A phase I/II clinical trial of enzyme replacement therapy in mucopolysaccharidosis II (Hunter Syndrome). Mol Gen Metab.

[Muenzeretal2009] Muenzer J., Wraith J.E., Clarke L.A., International Consensus Panel on Management Treatment of Mucopolysaccharidosis I. (2009). Mucopolysaccharidosis I: Management and treatment guidelines. Pediatrics.

[Munoz-Rojasetal2008] Muñoz-Rojas M.V., Vieira T., Costa R., Fagondes S., John A., Jardim L.B., Vedolin L.M., Raymundo M., Dickson P.I., Kakkis E. (2008). Intrathecal enzyme replacement therapy in a patient with mucopolysaccharidosis type I and symptomatic cord compression. Am J Med Genet.

[Nelson1997] Nelson J. (1997). Incidence of the mucopolysaccharidoses in Northern Ireland. Hum Genet.

[Nelsonetal2003] Nelson J., Crowhrst J., Carey B., Greed L. (2003). Incidence of the mucopolysaccharidoses in Western Australia. Am J Med Genet.

[NeufeldandMuenzer2001] Neufeld E.F., Muenzer J., Scriver C.R., Beaudet A.L., Sly W.S., Valle D. (2001). The mucopolysaccharidosis. The Metabolic and Molecular Bases of Inherited Disease.

[Ochiaietal2003] Ochiai T., Ito K., Okada T., Chin M., Shichino H., Mugishima H. (2003). Significance of extensive Mongolian spots in Hunter's syndrome. Br J Dermatol.

[Ouditetal2007a] Oudit G.Y., Butany J., Williams W.G., Clarke J.T., Iwanochko R.M. (2007a). Images in cardiovascular medicine. Left ventricular aneurysm associated with mucopolysaccharidosis type VI syndrome (Maroteaux-Lamy syndrome). Circulation.

[Ouditetal2007b] Oudit G.Y., Butany J., Williams W.G., Siu S.C., Clarke J.T., Iwanochko R.M. (2007b). Left ventricular aneurysm in a patient with mucopolysaccharidosis type VI (Maroteaux-Lamy syndrome): Clinical and pathological correlation. Cardiovasc Pathol.

[Pastoresetal2007] Pastores G.M., Arn P., Beck M., Clarke J.T., Guffon N., Kaplan P., Muenzer J., Norato D.Y., Shapiro E., Thomas J. (2007). The MPS I registry: Design, methodology, and early findings of a global disease registry for monitoring patients with mucopolysaccharidosis type I. Mol Genet Metabol.

[Pereiraetal2008] Pereira V.G., Martins A.M., Micheletti C., D'Almeida V. (2008). Mutational and oxidative stress analysis in patients with mucopolysaccharidosis type I undergoing enzyme replacement therapy. Clin Chim Acta.

[Petryetal2003] Petry M.F., Dieter T., Burin M., Giugliani R., Leistner S. (2003). Identification of a novel mutation in the ARSB gene that is frequent among Brazilian MPS VI patients. Genet Test.

[Petryetal2005] Petry M.F., Nonemacher K., Sebben J.C., Schwartz I.V., Azevedo A.C., Burin M.G., de Rezende A.R., Kim C.A., Giugliani R., Leistner-Segal S. (2005). Mucopolysaccharidosis type VI: Identification of novel mutations on the arylsulphatase B gene in South American patients. J Inherit Metab Dis.

[Poorthuisetal1999] Poorthuis B.J., Wevers R.A., Kleijer W.J., Groener J.E., de Jong J.G., van Weely S., Niezen-Koning K.E., van Diggelen O.P. (1999). The frequency of lysosomal storage diseases in The Netherlands. Hum Genet.

[Prasadetal2008] Prasad V.K., Mendizabal A., Parikh S.H., Szabolcs P., Driscoll T.A., Page K., Laksshminarayanan S., Allison J., Wood S., Semmel D. (2008). Unrelated donor umbilical cord blood transplantation for inherited metabolic disorders in 159 pediatric patients from a single center: Influence of cellular composition of the graft on transplantation outcomes. Blood.

[Randalletal2008] Randall D.R., Sinclair G.B., Colobong K.E., Hetty E., Clarke L.A. (2008). Heparin cofactor II-thombin complex in MPSI: A biomarker of MPS disease. Mol Genet Metab.

[Rogoyskietal1985] Rogoyski A., Czartoryska B., Kleijer W.J., Niermeijer M.F., Tronowska T.D., Gorska D., Polatynska-Krzyspiak B., Zaremba J. (1985). Postnatal and prenatal diagnosis of Maroteaux-Lamy syndrome. Acta Anthropogenet.

[Sanjurjo-Crespo2007] Sanjurjo-Crespo P. (2007). Mucopolysaccharidosis type II: Clinical aspects. Ver Neurol.

[Schumacheretal2008] Schumacher R.G., Brzezinska R., Schulze-Frenking G., Pitz S. (2008). Sonographic ocular findings in patients with mucopolysaccharidoses I, II and VI. Pediatr Radiol.

[Schwartzetal2007] Schwartz I.V.D., Ribeiro M.G., Mota J.G., Toralles M.B.P., Correia P., Horovitz D., Santos E., Monlleo I.L., Fett-Conte A.C., Sobrinho R.P. (2007). Titulo. Acta Paediatr Suppl.

[Sifuentesetal2007] Sifuentes M., Doroshow R., Hoft R., Mason G., Walot I., Diament M., Okazaki S., Huff K., Cox G.F., Swiedler S.J. (2007). A follow up study of MPS I patients treated with laronidase enzyme replacement therapy for 6 years. Mol Genet Metab.

[Solimanetal2007] Soliman O.I.I., Timmermans R.G.M., Nemes A., Vletter W.B., Wilson J.H., Ten Cate F.J., Geleijnse M.L. (2007). Cardiac abnormalities in adults with the attenuated form of mucopolysaccharidosis type I. J Inherit Metab Dis.

[Stabaetal2004] Staba S.L., Escolar M.L., Poe M., Kim Y., Martin P.L., Szabolcs P., Allison-Thacker J., Wood S., Wenger D.A., Rubinstein P. (2004). Cord blood transplants from unrelated donors in patients with Hurler's syndrome. N Engl J Med.

[Swiedleretal2005] Swiedler S.J., Beck M., Bajbouj M., Giugliani R., Schwartz I., Harmatz P., Wraith J.E., Roberts J., Ketteridge D., Hopwood J.J. (2005). Threshold effect of urinary glycosaminoglycans and the walk test as indicators of disease progression in a survey of subjects with Mucopolysaccharidosis VI (Maroteaux-Lamy syndrome). Am J Med Genet.

[Tanetal1992] Tan C.T., Schaff H.V., Miller J.R.F.A., Edwards W.D., Karpnes P.S. (1992). Valvular heart disease in four patients with Maroteaux-Lamy syndrome. Circulation.

[Tolaretal2008] Tolar J., Grewal S.S., Bjoraker K.J., Whitley C.B., Shapiro E.G., Charnas L., Orchard P.J. (2008). Combination of enzyme replacement and hematopoietic stem cell transplantation as therapy for Hurler syndrome. Bone Marrow Transplant.

[Turneretal1999] Turner C.T., Hopwood J.J., Bond C.S., Brooks D.A. (1999). Immune response to enzyme replacement therapy: 4-sulfatase epitope reactivity of plasma antibodies from MPS VI cats. Mol Genet Metab.

[Tuschletal2005] Tuschl K., Gal A., Paschke E., Kircher S., Bodamer O.A. (2005). Mucopolysaccharidosis type II in females: Case report and review of the literature. Pediatr Neurol.

[Vellodietal1997] Vellodi A., Young E.P., Cooper A., Wraith J.E., Winchester B., Meaney C., Ramaswami U., Will A. (1997). Bone marrow transplantation for mucopolysaccharidosis type I: Experience of two British centres. Arch Dis Child.

[VijayandWraith2005] Vijay S., Wraith J.E. (2005). Clinical presentation and follow-up of patients with the attenuated phenotype of mucopolysaccharidosis type I. Acta Paediatr.

[Wegrzynetal2007] Wegrzyn G., Tylki-Szymaska A., Liberek A., Piotrowska E., Jakóbkiewicz-Banecka J., Marucha J., Czartoryska B., Wegrzyn A. (2007). Rapid deterioration of a patient with mucopolysaccharidosis type I during interruption of enzyme replacement therapy. Am J Med Genet Part A.

[Weissteinetal2004] Weisstein J.S., Delgado E., Steinbrach L., Hart K., Packman S. (2004). Musculoskeletal manifestations of Hurler Syndrome: Long-term follow-up after bone marrow transplantation. J Pediatr Orthop.

[Wraith2001] Wraith J.E. (2001). Enzyme replacement therapy in mucopolysaccharidosis type I: Progress and emerging difficulties. J Inher Metab Dis.

[Wraith2005] Wraith J.E. (2005). The first 5 years of clinical experience with laronidase enzyme replacement therapy for mucopolysaccharidosis I. Expert Opin Pharmacother.

[Wraithetal2004] Wraith J.E., Clarke L.A., Beck M., Kolodny E.H., Pastores G.M., Muenzer J., Rapoport D.M., Berger K.I., Swiedler S.J., Kakkis E.D. (2004). Enzyme replacement therapy for mucopolysaccharidosis I: A randomized, double-blinded, placebo-controlled, multinational study of recombinant human α-L-iduronidase (laronidase). J Pediatr.

[Wraithetal2007] Wraith J.E., Beck M., Lane R., van der Ploeg A., Shapiro E., Xue Y., Kakkis E.D., Guffon N. (2007). Enzyme replacement therapy in patients who have mucopolysaccharidosis I and are younger than 5 years: Results of a multinational study of recombinant human α-L-iduronidase (laronidase). Pediatrics.

[Wraithetal2008] Wraith J.E., Scarpa M., Beck M., Bodamer A.O., De Meirleir L., Guffon N., Lund A.M., Malm G., Vand der Ploeg A., Zeman J. (2008). Mucopolysaccharidosis type II (Hunter syndrome): A clinical review and recommendations for treatment in the era of enzyme replacement therapy. Eur J Pediatr.

[Zareba2007] Zareba G. (2007). Idursulfase in Hunter Syndrome treatment. Drugs Today (Barc).

