# Supplementary material for: Mucopolysaccharidosis I, II, and VI: Brief review and guidelines for treatment
Source: Genet Mol Biol. 2010 Dec 1;33(4):589–604. doi: 10.1590/S1415-47572010005000093 (PMC3036139; doi:10.1590/S1415-47572010005000093)
Supplement: Supplementary file 1 — Note about the steps followed for the drafting of this document [file gmb-33-4-589-suppl1.pdf]

# Note about the steps followed for the drafting of this document

For the drafting of this document, the following steps were performed:

## 1) Collection of Initial Data and Drafting of the Preliminary Document:

This phase was carried out at the Medical Genetics Service [SGM] of the Porto Alegre Clinical Hospital [HCPA (Hospital de Clínicas de Porto Alegre)]. Six members of the SGM/HCPA mucopolysaccharidosis staff, suggested by Prof. Roberto Giugliani, were invited and agreed to take part in the following teams:

- MPS I – Verónica Muñoz and Andressa Federhen
- MPS II – Louise Pinto and Taiane Vieira
- MPS VI – Ana Cecília Azevedo and Osvaldo Artigalás

A systematic review of the literature along with the PubMed and Bireme databases was initially performed. The following keywords were used for the search:

• Mucopolysaccharidoses
• Mucopolysaccharidosis I
    Hurler syndrome
    Scheie syndrome
    Hurler-Scheie syndrome
• Mucopolysaccharidosis II
    Hunter syndrome
• Mucopolysaccharidosis VI
    Maroteaux-Lamy syndrome
  Enzyme replacement therapy
  Natural history
  Treatment
  Clinical trial
  Surveys & Registries

Also included were articles accepted for publication and articles that contained information relevant to the drafting of the document, even if the keywords did not appear in them. From the articles collected, a text on MPS I, MPS II, and MPS VI was drafted following the outline below:

### a) Brief summary on each disease, covering:

– clinical picture
– natural course, including most common complications
– inheritance and incidence
– biochemical and molecular defect
– diagnostic process
– conventional treatments
– prevention (detection of carriers, genetic counseling, and prenatal diagnosis)

### b) Enzyme Replacement Therapy

– history (preclinical studies)
– summary of clinical trials (phases I, II, and III)
– summary of the experience to date
– guidelines for ERT:
– indications
– usage regimen (frequency, dose, infusion time)
– premedication
– preparation of the drug
– instructions regarding filters
– adverse reactions – conduct
– adverse reactions – pharmacovigilance
– clinical routines for the patient receiving ERT
– periodic evaluations
– criteria for discontinuation of treatment
– effect of discontinuation of treatment

## 2) Evaluation by Brazilian Specialists and Production of a Consolidated Document:

After the preliminary document was drafted, it was sent by e-mail to a group of Brazilian professionals from all the five regions of the country with experience in the matter, including representatives of medical societies with an interest in the area, in order for them to become acquainted with it.

A workshop was then organized which these specialists were invited to, for a general discussion of the document. This meeting was held in Caxias do Sul, on May 16, 17, and 18, 2008, with over 40 members of the group present.

Each topic of the document was discussed and the suggestions were recorded by the main writing team and by a medical writing specialist who was added to the group. A consolidated document was then prepared and sent to the group members, who made further suggestions, which were incorporated into this version.

## 3) Prospects for Revision of the Document

Throughout the process of drafting this document, various suggestions for improving it came up, which will be the subject of a new round of discussion, aiming to the production of a second version. Among these are:

a) drafting of protocols for the diagnosis of patients with MPS;
b) drafting of protocols for the treatment of these conditions;

c) drafting of clinical and laboratory protocols for patient evaluation and follow-up;
d) drafting of a checklist with minimum criteria and recommended criteria for the setting-up of an enzyme replacement therapy infusion center for MPS;
e) establishment of objective criteria for discontinuation of treatment;
f) inclusion of a directory of professionals and departments experienced in the diagnosis and treatment of these diseases in different locations in Brazil;
g) discussion and drafting of a sample Informed Consent Form for use in specific cases that do not meet the usual inclusion criteria;
h) a general review, possible adoption of a new format, and updating of the document in light of new evidence that has appeared in the literature and the growing experience of the professionals participating in the group.
